# Does the Angiotensin-converting enzyme (ACE) gene insertion/deletion polymorphism modify the response to ACE inhibitor therapy? – A systematic review

**DOI:** 10.1186/1468-6708-6-16

**Published:** 2005-10-24

**Authors:** Madlaina Scharplatz, Milo A Puhan, Johann Steurer, Annalisa Perna, Lucas M Bachmann

**Affiliations:** 1 Horten Centre for patient oriented research, University of Zurich, Switzerland; 2 Mario Negri Institute for Pharmacological Research, Clinical Research Center for Rare Diseases, "Aldo e Cele Daccò" Villa Camozzi, Bergamo, Italy; 3 Division of Epidemiology and Biostatistics, Department of Social and Preventive Medicine, University of Bern, Switzerland

**Keywords:** Angiotensin-converting enzyme, pharmacogenetics, ACE I/D polymorphism

## Abstract

**Background:**

Pharmacogenetic testing to individualize ACE inhibitor therapy remains controversial. We conducted a systematic review to assess the effect modification of the insertion/deletion (I/D) polymorphism of the ACE gene on any outcome in patients treated with ACE inhibitors for cardiovascular and/or renal disease.

**Methods:**

Our systematic review involved searching six electronic databases, then contacting the investigators (and pharmaceutical industry representatives) responsible for the creation of these databases. Two reviewers independently selected relevant randomized, placebo-controlled trials and abstracted from each study details on characteristics and quality.

**Results:**

Eleven studies met our inclusion criteria. Despite repeated efforts to contact authors, only four of the eleven studies provided sufficient data to quantify the effect modification by genotypes. We observed a trend towards better response to ACE inhibitors in Caucasian DD carriers compared to II carriers, in terms of blood pressure, proteinuria, glomerular filtration rate, ACE activity and progression to end-stage renal failure. Pooling of the results was inappropriate, due to heterogeneity in ethnicity, clinical domains and outcomes.

**Conclusion:**

Lack of sufficient genetic data from the reviewed studies precluded drawing any convincing conclusions. Better reporting of genetic data are needed to confirm our preliminary observations concerning better response to ACE inhibitors among Caucasian DD carriers as compared to II carriers.

## Background

Angiotensin-converting enzyme (ACE) inhibitors have become a cornerstone in the management of patients with cardiovascular disorders [1]. A large number of trials have demonstrated important clinical benefits for this class of drugs in patients with arterial hypertension [2], heart failure [3-6], diabetic and non-diabetic nephropathy [7-11] and in patients who have undergone renal transplantation [12,13]. Despite the generally positive effects of ACE inhibitors, response to equivalent doses of these drugs varies considerably among individuals [14]. As an example, up to one third of patients with congestive heart failure may not tolerate or respond to ACE inhibitor therapy [15,16].

In the late 1980s, researchers began investigating genetic factors to determine the origins of inter-individual variability in patients' responses to ACE inhibitor therapy. Rigat et al. [17] identified the insertion/deletion (I/D) polymorphism of the ACE gene, which is based on the presence (insertion) or absence (deletion) of a 287-bp element on intron 16 on chromosome 17. They noted that this polymorphism accounted for about 47% of the phenotypic variance for serum ACE levels. This led to the hypothesis that the I/D polymorphism may influence the effect of ACE inhibitors on clinical outcomes.

The ACE I/D polymorphism has received much attention since its discovery, and many pharmacogenetic studies have been conducted in different patient groups to assess its effect modification. Debate, however, continues regarding the extent and direction of its effect modification [8,14,18]. The objective of our study was to systematically review all randomised, placebo-controlled trials that had evaluated to what extent the ACE gene insertion/deletion polymorphism influences treatment effects of ACE inhibitors on any surrogate and on any clinically relevant parameters in patients with cardiovascular diseases, diabetes, renal transplantation and/or renal disease.

## Methods

A previous publication details our study methodology [19].

### Search Strategy

We performed extensive literature searches in (Pre-) MEDLINE (DataStar^® ^version Cary North Carolina from inception to 2005), EMBASE (DataStar^® ^version from inception to 2005, Cary North Carolina), Biosis (Ovid^® ^version "Previews 1989 to 2005", New York), the Cochrane Controlled Trials Register (CCTR <2rd Quarter 2005>, Oxford, United Kingdom) and the Science Citation Index. In collaboration with an information specialist, we carried out a preliminary literature search in Medline to design the final search strategy [19]. We used the following final search terms "peptidyl-dipeptidase A," "dipeptidyl-carboxypeptidase-inhibitor," "ACE inhibitor," "genetics," "pharmacogenetic" and "polymorphism" without language restriction. The last search was performed in July, 2005.

We also contacted authors who had published pharmacogenetic analyses in the area of cardiovascular disease, diabetes, renal transplantation and/or renal diseases, as well as relevant collaborative review groups of the Cochrane Collaboration and pharmaceutical companies for additional published or unpublished data. Finally, we reviewed bibliographies of all included studies to identify additional relevant articles, using the "related articles" function of PubMed and the citation index of ISI Web of Science by entering all studies included in the review.

### Inclusion criteria

Two reviewers (MS, MAP) independently assessed all obtained titles and abstracts stored in Reference Manager^® ^files (Professional Edition Version 11, ISI ResearchSoft, Berkeley, California) and ordered the full text of all potential articles. The two reviewers then examined the full texts of all retrieved citations and included studies if they 1) were randomised controlled trials comparing ACE inhibitors to placebo or to a non-active treatment 2) included patients with heart failure, primary and secondary hypertension, coronary artery disease, diabetic nephropathy, primary nephropathy and patients who had undergone renal transplantation and 3) had determined the insertion/deletion polymorphism.

### Data extraction and quality assessment

Two reviewers independently abstracted the data and assessed the quality of the included studies. We corrected discordant items based on obvious reading errors and resolved through consensus discordance based on real differences in interpretation. A third reviewer (LMB) resolved any remaining discrepancies.

We used a pre-designed and pilot-tested data extraction form [19] and recorded details on study design, treatments, patients, pharmacogenetic tests, outcome parameters and results. We also focused our efforts on obtaining additional unpublished data on genetic test information and effect measures from authors of included studies. We sent our requests and subsequent reminders for these data to the first and last authors. To assess internal validity of the included studies, we utilized a detailed list of quality items [20], adding other items that we considered to be important for pharmacogenetic studies (e.g., blinding of laboratory assessor of study outcomes, blinding of outcome assessor for genotypes and blinding of treatment provider for genotypes).

### Methods of analysis and synthesis

Results of the data extraction and assessment of study validity were summarized in structured tables. In comparing the intervention group (ACE inhibitor) with control groups (placebo or no active control), treatment effects for each genetic subgroup (DD, DI and II) were assessed by calculating mean differences for continuous outcomes and relative risks for dichotomous outcomes. We also calculated mean differences and relative risks between intervention and control groups for the whole study population, including all three genetic subgroups (DD/DI/II), in order to provide an overall treatment effect of the study. All statistical analyses were performed using the Stata^® ^statistical software package (StataCorp. 2004. Stata^® ^Statistical Software: Release 8.2 College Station, Texas, USA).

## Results

**Figure 1:**
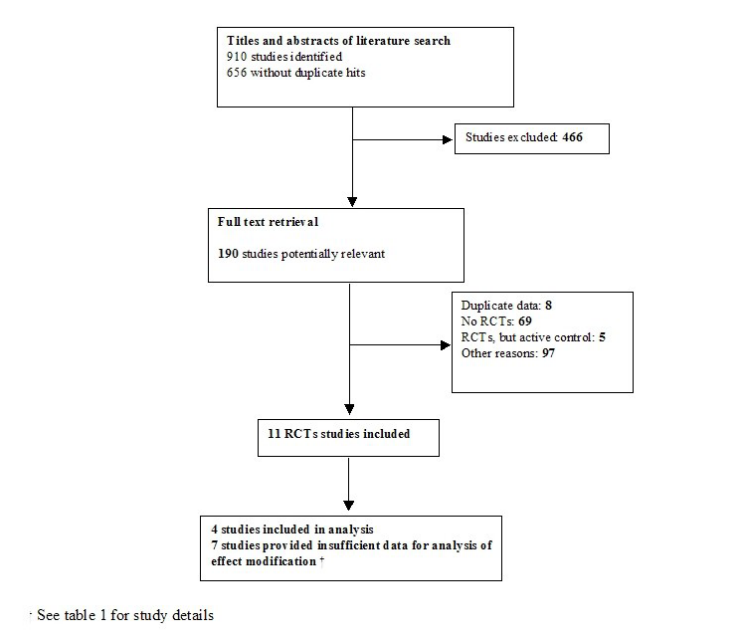
**Study flow from identification to final inclusion of studies. **We identified 656 studies from which 11 studies eventually met the inclusion criteria.

Figure 1 summarizes the selection process for the 656 identified abstracts. The eleven placebo-controlled, randomized clinical trials (RCTs) that met the final inclusion criteria involved post-myocardial infarction participants as well as subjects with congestive heart failure (n = 3), arterial hypertension (n = 1), chronic proteinuric nephropathies (n = 1), diabetes (n = 2) and patients who had undergone coronary stent implantation or coronary angioplasty (n = 3) or renal transplantation (n = 1). Only three trials reported sufficient data [21-23] and the authors of one trial sent additional requested data [24] for the analysis of effect modification. Despite repeated efforts to obtain additional unpublished data from the remaining trials, [23,25-31], we were unable to secure this information.

Table 1 summarizes the methodological quality of all included trials. Disagreement on quality assessment was resolved through consensus. In general, the quality of trials was poor to moderate. Trials scored poorly for blinding, which was considered only for interventions but not for genotypes. The four trials included in the analysis of the effect modification were of moderate methodological quality. All four studies controlled for imbalances of baseline characteristics between genotypes, whereas only three studies [21,23,24] controlled for co-interventions that might affect study outcomes.

**Table 1:** Internal validity of included randomized controlled trials [20]

	*Homogenous study group*	*Blinding of outcome assessor for genotype*	*Blinding of outcome assessor for treatment*	*Blinding of laboratory assessor for outcome*	*Blinding of treatmen t provider (doctor) for genotype*	*Blinding of treatment treatment*	*Blinding of patients for treatment*	*Check to what extent blinding was successful*	*Registration of loss to follow-up*	*Random allocation (description of procedure)*	*Treatment allocation concealed*
Hernandez 2000 [25]	**+**	**+**	**+**	**-**	**-**	**-**	**+**	**-**	**+**	**-**	**-**
Hingorani 1997 [26]	**+**	**-**	**-**	**-**	**-**	**-**	**-**	**-**	**-**	**+/-**	**-**
Kventy 2000 [27]	**+/-**	**-**	**-**	**-**	**-**	**+**	**+**	**-**	**+**	**-**	**-**
Meurice 2001 [28]	**+/-**	**-**	**-**	**-**	**-**	**+**	**+**	**-**	**+**	**-**	**+/-**
Okamura 1999 [21]	**-**	**-**	**-**	**-**	**-**	**-**	**-**	**-**	**+**	**-**	**-**
Okumura 2002 [29]	**+**	**-**	**-**	**+**	**-**	**-**	**-**	**-**	**+/-**	**-**	**-**
Pedersen 1997 [30]	**-**	**+**	**+**	**+**	**-**	**+**	**+**	**-**	**+**	**+/-**	**-**
Penno 1998 [23]	**+/-**	**-**	**-**	**+/-**	**-**	**+**	**+**	**-**	**+**	**+**	**+**
Perna 1999 [24]	**+/-**	**-**	**-**	**-**	**-**	**+**	**+**	**-**	**-**	**+**	**+**
Pinto1995 [31]	**-**	**+**	**+**	**+**	**-**	**+**	**+**	**-**	**-**	**-**	**-**
Van Geel 2003 [22]	**+**	**-**	**-**	**-**	**-**	**-**	**+**	**-**	**-**	**+/-**	**-**

	*Methods for dealing with missing values*	*Compliance checked*	*Analysis of CI*	*Control for possible clinical and other confounders between genotypes*	*Control of co- interventions that bear on outcome for each genotype*	*Per protocol analysis*	*Intention to treat analysis*				

Hernandez 2000 [25]	**-**	**+/-**	**-**	**+**	**-**	**-**	**+**				
Hingorani 1997 [26]	**+/-**	**-**	**-**	**+**	**-**	**+/-**	**-**				
Kventy 2000 [27]	**-**	**-**	**-**	**-**	**-**	**-**	**-**				
Meurice 2001 [28]	**+**	**+**	**-**	**-**	**+**	**+**	**+**				
Okamura1999 [21]	**-**	**-**	**-**	**+/-**	**+/-**	**-**	**-**				
Okumura 2002 [29]	**-**	**-**	**-**	**-**	**-**	**+**	**-**				
Pedersen 1997 [30]	**-**	**+/-**	**+/-**	**-**	**-**	**+/-**	**-**				
Penno 1998 [23]	**+/-**	**+/-**	**+**	**+/-**	**+/-**	**+/-**	**+**				
Perna 1999 [24]	**-**	**-**	**-**	**+/-**	**-**	**-**	**+**				
Pinto 1995 [31]	**-**	**+**	**-**	**-**	**-**	**+/-**	**+**				
Van Geel 2003 [22]	**-**	**-**	**-**	**+/-**	**-**	**+/-**	**-**				

***+ ****: Fulfilled; ****+/-****: Partially fulfilled; ****-****: Not fulfilled or no information provided*

### Patient characteristics of included studies

Details about the individual trials and participant profiles are provided in Table 2. Mean age of patients with cardiovascular disease and renal disease plus diabetes was 66 and 48 years, respectively. Nine of the 11 studies included Caucasians, while two studies investigated Asian populations.

**Table 2:** Study characteristics of RCTs

**Study**	**Population**	**ACE intervention Control intervention**	**Mean follow up**	**Additional interventions**	**Outcomes**
Perna 1999 [24]	212 (87 DD/ 99DI /26 II) **Caucasians**, mean age of 50 years, with **non- diabetic **proteinuric chronic nephropathies: (urinary protein excretion> 1 g/24 h for last 3 months and creatinine clearance of 20 – 70 mL/min/1.73m2)	Ramipril 1.25, increased to 2.5 or 5 mg/d Placebo or conventional treatment	30.3 months	Conventional treatment for chronic nephropathy	***Blood pressure, proteinuria, glomerular filtration rate, end stage renal disease***
Van Geel 2003 [22]	86 (20 DD/ 43 DI/ 23 II) **Caucasians**, mean age of 62 years, undergoing **elective coronary artery bypass graft surgery**; able to take the study drug for at least seven days before surgery	Quinapril 40 mg/d Placebo	12 months	Aspirin, coumarin-derivatives (anticoagulants), β- blocker, Ca^+2 ^channel, nitrates	***Plasma ACE activity***
Okamura 1999 [21]	97 (16 DD/26 DI/36 II) **Asians**, mean age of 63 years, with stable angina pectoris undergoing **percutaneous transluminal coronary angioplasty**	Imidapril 5 mg/d Placebo	3–6 months	Aspirin and warfarin	***Minimal lumen diameter of coronary artery (net gain (mm), diameter stenosis %, late loss (mm) and loss index)***
Penno 1998 [23]	N = 530 (137 DD/296 DI /77 II) **Caucasians, **mean age of 53, **diabetes **with normo- and microalbuminuria, diastolic blood pressure < 90 mmHg and > 75 mmHg, systolic blood pressure < 155 mmHg	Lisinopril, 10–20mg/d Placebo	24 months	Glycaemic control	***Proteinuria***
Hernandez 2000 [25]	N= 52 (25 DD/DI and 17 II) **Caucasians**, mean age of 47, patients with **renal transplantation**, Stable renal function (creatinine clearance < 2.5 mg/dL for more than 6 months), absence of renal artery stenosis or chronic allograft nephropathy, no renovascular hypertension, absence of proteinuria	Lisinopril, 10 mg/d Placebo	12 months	Antithymocyte globulin, prednisone, cyclosporine, azathioprine	***Serum creatinine, left ventricular ejection fraction, left ventricular isovolumetric relaxation time, left ventricular mass index***
Hingorani 1997 [26]	N = 125 (37 DD/70 DI/17 II) **Caucasians**, mean age of 54, with untreated **essential hypertension **>160/90 (mainly primary care referrals	Captopril (50 mg/d), Enalapril (10 mg/d), Lisinopril (10 mg/d), Perindopril (4 mg/d) Placebo	4 weeks	Ca^+2^-channel blocker	***Blood pressure***
Kventy 2000 [27]	N = 57 (17/DD/ 24 DI /16 II) **Caucasians**, mean age of 45, with **diabetes **> 5 years, no microalbuminuria < 20 ug/min, albumin creatinine ratio < 2.5 mg/mmol, normal serum creatinine and urine sediment	Perindopril, 4 mg/d Placebo	24 months	None	***Albumin/creatinine ratio, blood pressure, glomerular filtration rate***
Meurice 2001 [28]	N = 91 (only DD) **Caucasians**, mean age of 85, after coronary **stent implantation**	Quinapril 40 mg/d Placebo	6 months	Aspirin (75–300 mg for 6 months), ticlopidine (500mg for 1 months)	***Minimal luminal diameter of coronary artery (restenosis)***
Pedersen 1997 [30]	N = 56 (14 DD, 26 DI,16 II) **Caucasians**, mean age of 68, **after acute myocardial infarction **and moderate to severe **left ventricular dysfunction **(2–6 days after myocardial infarction)	Trandolapril, 4 mg/d Placebo	12 months	None	***Tissue-type plasminogen activator [30]32 and plasminogen activator inhibitor (PAI) ACE activity***
Pinto 1995 [31]	N = 96 (34 DD/DI and 62 II) **Caucasians **with first **anterior myocardial infarction**, treated with thrombolytic therapy (streptokinase intravenously)	Captopril 75 mg/d Placebo	12 months	Streptokinase administration (1500000U) Nitrates, Ca+2 channel blocker, β- blocker, aspirin, diuretics	***End-systolic and end-diastolic volume***
Okumura 2002 [29]	N = 100 (21 DD/22 DI/49 II) **Asians**, mean age of 64, **after coronary artery stent implantation**	Quinapril 10–20 mg/d Placebo	6 months	Ca^+2 ^channel blocker, β- blocker, nitrates, aspirin, ticlopidine	***Minimal lumen diameter of coronary artery (restenosis)***

Of the studies included in the analysis of the effect modification, three had enrolled Caucasians with non-diabetic chronic nephropathies [24], insulin-dependent diabetes mellitus [23] or patients undergoing elective coronary artery bypass graft surgery [22]. One study included Asians who had undergone percutanous transluminal coronary angioplasty [21]. In all four studies, ACE inhibitors were assigned as add-on therapy to slow disease progression.

### Comparisons between genotypes across clinical domains

**Figure 2:**
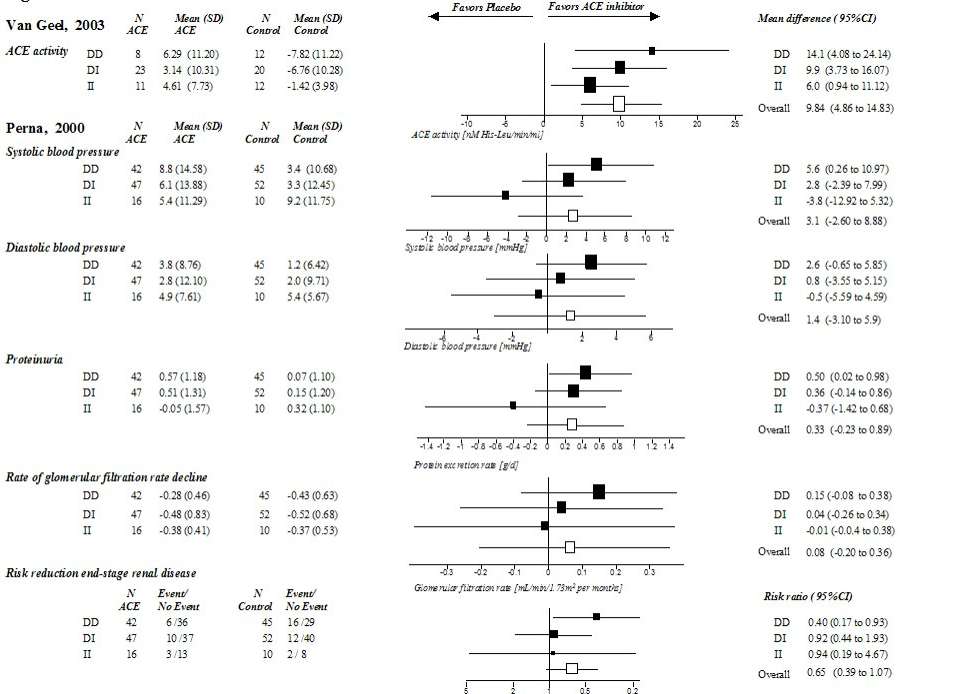
**Comparison of treatment effects between genotypes in Caucasians **(results of the study of Van Geel [22] and Perna [24] on different outcomes). Treatment effects for each genotype (DD/DI/II) and overall treatment effects are presented as mean differences (from baseline to follow up) or relative risks with 95% confidence intervals.

The studies of Perna [24] and Van Geel [22], which were based on previously published trials [32-34], provided sufficient data to assess the overall ACE inhibitor effects on different outcomes. Perna et al. assessed systolic and diastolic blood pressure, proteinuria, glomerular filtration rate and end-stage renal disease (defined as need for dialysis), and Van Geel et al. assessed the plasma ACE activity for each genotype. In these two studies, which included Caucasians with the DD genotype, ACE inhibitors reduced systolic blood pressure more effectively (mean difference 5.6 mmHg, 95% confidence intervals [0.96 to 10.97]) compared to the overall treatment effect (3.1 mmHg [-2.60 to 8.88]), while in II carriers, ACE inhibitors increased systolic blood pressure (-3.8 mmHg [-12.92 to 5.32]) compared to placebo (Figure 2). Effect modification with benefit for DD carriers and treatment failure for II carriers was also present for diastolic blood pressure, decline of glomerular filtration rate, ACE activity and progression to end-stage renal disease. Differences between genotypes failed to achieve statistical significance. Details on baseline characteristics and differences in treatment effects across genotypes are shown in Table 3. The risk reduction of the incidence of end-stage renal disease was 22% (4% to 39%) for DD carriers compared to 2% (-14% to 18%) for DI carriers and 1% (-21% to 41%) for II carriers (see Figure 2).

**Table 3:** Data of effect modification for continuous clinical outcomes

***Outcome Study***	***Genotype***	***N (ACE)***	***Baseline***	***Follow up***	***Mean (SD) ACE inhibitor***	***N (Control)***	***Baseline***	***Follow up***	***Mean (SD) Control group***	***Mean difference (estimated SD)***	***Overall effect (weighted mean and SD)***
*Reduction in ACE activity (U/I)*											
Van Geel [22]	DD	8	31†	25†	6.29 (11.20)	12	27†	35†	-7.82 (11.22)	**14.11** **(11.2)**	**9.84** **(0.83)**
	DI	23	25.5†	22†	3.14 (10.31)	20	23†	20†	-6.76 (10.28)	**9.9** ** (10.3)**	
	II	11	18†	13†	4.61 (7.73)	12	18†	20†	-1.42 (3.98))	**6.03** ** (6.1)**	
*Reduction in end-systolic blood pressure (mmHg)*											
Perna [24]	DD	42	147.8 (19.1)	139.8 (15.1)	8.8 (14.58)	45	146 (17.7)	142.8 (13.9)	3.2 (10.68)	**5.6 ** **(12.8)**	**3.1** ** (12.8)**
	DI	47	145.1 (18.9)	139 (17.5)	6.1 (13.88)	52	146.5 (17.2)	143.2 (12.72)	3.3 (12.45)	**2.8** ** (13.08)**	
	II	16	145.1 (18.4)	139.7 (17.8)	5.4 (11.29)	10	153.2 (15.9)	144 (14.7)	9.2 (11.75)	**-3.8** ** (11.85)**	
*Reduction in end-diastolic blood pressure (mmHg)*											
Perna [24]	DD	42	89.9 (11)	86.2 (6.9)	3.8 (8.76)	45	88.6 (10.2)	87.4 (7.6)	1.2 (6.42)	**2.6 ** **(7.8)**	**1.4** ** (9.1)**
	DI	47	88.9 (13.9)	86.1 (9.64)	2.8 (12.10)	52	90.6 (12.6)	88.6 (8.86)	2.0 (9.7)	**0.8** ** (10.9)**	
	II	16	90.8 (11.6)	85.3 (5.8)	4.9 (7.61)	10	97.5 (6.1)	92.1 (6.25)	5.4 (5.67)	**-0.5** ** (7.0)**	
*Reduction of proteinuria (g/24 h)*											
Perna [24]	DD	42	2.85 (1.69)	2.28 (1.5)	0.57 (1.18)	45	2.8 (1.7)	2.73 (1.4)	0.07 (1.10)	**0.5** ** (1.1)**	**0.33 ** **(1.2)**
	DI	47	3.3 (2.2)	2.97 (1.97)	0.51 (1.31)	52	3.8 (2.5)	3.65 (1.9)	0.15 (1.20)	**0.36** ** (1.2)**	
	II	16	3.45 (2.37)	3.41 (3.3)	0.05 (1.57)	10	3.4 (1.7)	3.08 (0.32)	0.32 (1.15)	**-0.37** ** (1.4)**	
*Decline of glomerular filtration rate (per months)*											
Perna [24]	DD	42	44.2 (19.1)	n.a.	-0.28 (0.46)	45	40.2 (17)	n.a.	-0.43 (0.63)	**0.15** ** (0.54)**	**0.08** ** (0.63)**
	DI	47	45.3 (20.7)	n.a.	-0.48 (0.83)	52	40.6 (17.1)	n.a.	-0.52 (0.68)	**0.04 ** **(0.75)**	
	II	16	47.3 (23.5)	n.a.	-0.38 (0.41)	10	47.8 (20.5)	n.a.	-0.37 (0.53)	**-0.01** ** (0.43)**	
*Albumin excretion rate(μg/min)*											
Penno [23]	DD	71	8.1*	17.1†	9.00†	66	8.1*	17.2†	9.78†	**-0.78**	**-7.33**
	DI	154	7.6*	17.4†	9.80†	142	7.6*	19.1†	11.5†	**-1.70**	
	II	29	9.2*	16.2†	7.00†	48	9.2*	24.3†	15.11†	**-8.11**	
*Net gain (mm)*											
Okamura [21]	DD	9	0.64 (0.27)	1.09 (0.66))	0.45 (0.23)	7	0.49 (0.27)	1.64 (0.96)	1.16 (0.26)	**-0.71** ** (0.2)**	**0.03 ** **(0.28)**
	DI	13	0.58 (0.32)	1.62 (0.79)	1.04 (0.24)	13	0.53 (0.18)	1.42 (0.97)	0.89 (0.26)	**0.15** ** (0.3)**	
	II	10	0.55 (0.25)	1.82 (0.69)	1.27 (0.21)	26	0.65 (0.16)	1.42 (0.54)	0.77 (0.18)	**0.5** ** (0.2)**	
*Late Loss (mm)*											
Okamura [21]	DD	9	2.43 (0.54)	1.09 (0.66))	1.34 (0.23)	7	2.46 (0.57)	1.64 (0.96)	0.82 (0.24)	**-0.52** ** (0.67)**	**0.15** ** (0.74)**
	DI	13	2.58 (0.55)	1.62 (0.79)	0.96 (0.21)	13	2.32 (0.49)	1.42 (0.97)	0.90 (0.23)	**-0.06** ** (0.79)**	
	I^24^I	10	2.44 (0.51)	1.82 (0.69)	0.62 (0.16)	26	2.65 (0.25)	1.42 (0.54)	1.23 (0.16)	**0.61** ** (0.75)**	
*Loss index (% at follow up)*											
Okamura [21]	DD	9	n.a.	n.a.	0.78 (0.13)	7	n.a.	n.a.	0.42 (0.12)	**-0.36** ** (0.36)**	**0.03** ** (0.39)**
	DI	13	n.a.	n.a.	0.53 (0.12)	13	n.a.	n.a.	0.49 (0.13)	**-0.04** ** (0.45)**	
	II	10	n.a.	n.a.	0.38 (0.09)	26	n.a.	n.a.	0.63 (0.08)	**0.25** ** (0.37)**	
*Diameter stenosis*											
Okamura [21]	DD	9	n.a.	n.a.	57.4 (23.4)	7	n.a.	n.a.	40.08 (26.4)	**-0.17** ** (0.25)**	**0.03** ** (0.39)**
	DI	13	n.a.	n.a.	43.8 (25.2)	13	n.a.	n.a.	45.1 (33.9)	**0.01 ** **(0.30)**	
	II	10	n.a.	n.a.	34.3 (19.9)	26	n.a.	n.a.	48.2 (30.6)	**0.14** ** (0.28)**	

Values are presented as means and SD; n.a.: not available; * indicates combined baseline data for intervention and control group; † indicates extracted values of figures (no SD)

The study of Penno et al. [23] was also based on a previously published trial [11]. Caucasian patients with insulin-dependent diabetes mellitus and normoalbuminuria (83% in DD, 87% in DI and 81% in II) at baseline showed comparable treatment effects from ACE inhibitors between genotypes within the first 12 months. Thereafter, II carriers had an enhanced response to ACE inhibitors with regard to reduction of albumin excretion rate (8.1 μg/min) compared to DI (1.7μg/min) and DD carriers (0.8μg/min). Patients with II genotypes also exhibited the largest benefit in terms of progression from normoalbuminuria to micro- or macroalbuminuria (Risk ratio 0.36 [0.05 to 2.74]), whereas in DD carriers, ACE inhibitors tended to have a negative effect (risk ratio of 1.18 [0.33 to 4.20]). There were, however, baseline imbalances in important prognostic variables between genotypes. Compared to DI and DD carriers, II carriers had pronounced albuminuria at baseline, with placebo group participants experiencing the greatest progression of albumin excretion rate.

**Figure 3:**
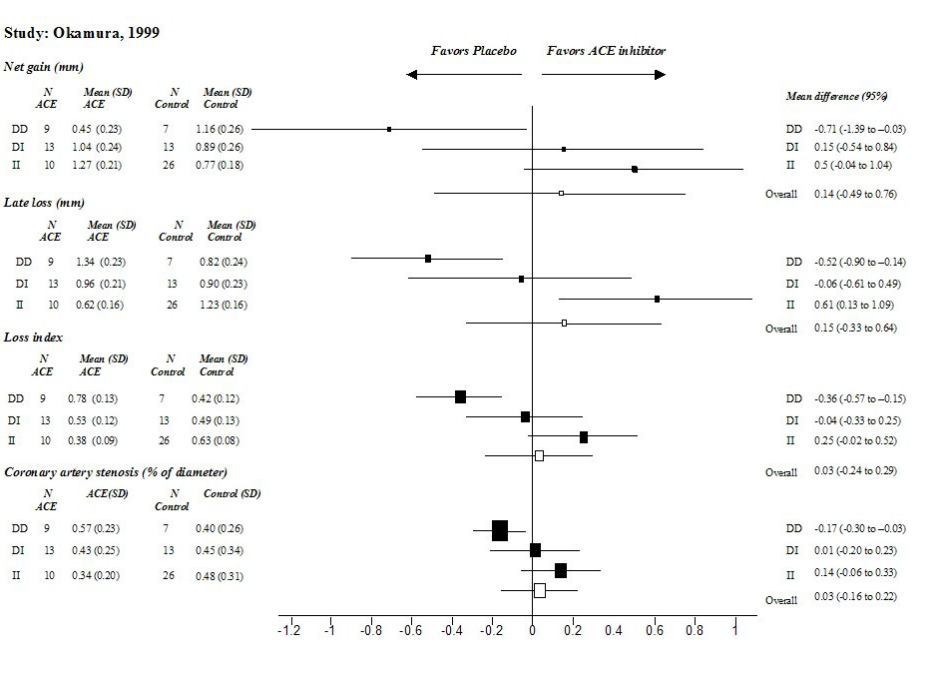
**Comparison of treatment effects between genotypes in Asians **(results of the study of Okamura [21] on differences in minimal luminal diameter). Effects for each genotype (DD/DI/II) and the overall treatment effect are presented as mean differences (from follow-up to baseline) with 95% confidence intervals.

In the study of Okamura [21], which included a Japanese population, only the II subgroup had an enhanced response to ACE inhibitors (manifested by prevention of restenosis, as defined by most indexes after percutaneous transluminal coronary angioplasty). Otherwise, the overall treatment effects were not significant (Figure 3). II carriers showed 1) an increased net gain in minimal lumen diameter of 0.5 mm (-1.04 to 1.04) compared to the overall effect of 0.14 mm (-0.49 to 0.76), 2) a higher percentage of change in diameter stenosis 0.14 (-0.06 to 0.33) compared to the overall effect of 0.03 (-0.16 to 0.22), 3) an improved late loss of lumen diameter of 0.61 mm (0.13 to 1.09) compared to the overall effect of 0.15 mm (-0.33 to 0.64) and 4) a better loss index (the ratio of late loss to acute gain) of 0.25 (-0.02 to 0.52) compared to the overall effect of 0.03 (-0.24 to 0.29). In the DD subgroup, ACE inhibitors showed a negative effect on changes in minimal lumen diameter of coronary arteries. Thus, Japanese DD carriers did not benefit from ACE inhibitor therapy, while DI carriers demonstrated moderate responses and II carriers showed the greatest treatment response.

## Discussion

Based on our systematic review, evidence quantifying the extent of effect modification related to the I/D polymorphism is sparse. We did note a trend towards better response to ACE inhibitor therapy in DD Caucasians as compared to II carriers, who seemed not to benefit.

The strengths of this review include the comprehensive literature search and strict adherence to systematic review methodology. We restricted our analyses to trials with placebo controls, as studies without a placebo control group do not allow for estimation of the ACE inhibitor effect and are likely to be confounded [35,36].

Although we identified 11 randomised controlled trials that assessed differences in treatment effects among genotypes, only the results of four trials studying 925 patients contributed to our analyses. The authors of the remaining seven studies that included 577 patients did not provide data about genetic subgroups in the intervention and control arms [26-28,30]. Others presented combined results for two different genotypes [25,29,31]. We made substantial efforts to contact the authors of these seven trials as well as researchers known to be active in the field of pharmacogenetics, but we did not succeed in receiving additional unpublished data. Thus, our analysis might be subject to publication bias. While publication bias is a common problem in systematic reviews, the situation might be aggravated in reviews of genetic data. Reporting of genetic data is in general poor and most "negative" results of association studies do not even reach conference proceedings [37].

Despite an overall lack of evidence, some of our findings merit attention. For example, the second largest study of Caucasians with chronic nephropathies [24] showed a consistent trend towards a beneficial effect for various surrogate and clinical outcomes, whereas II carriers appeared to be unresponsive to treatment. Comparable results have been observed in the reduction of plasma ACE activity for patients after coronary artery bypass surgery [22]. On the other hand, the findings of the largest study [23], including diabetic patients with normo- or microalbuminuria, yielded conflicting results to the above-discussed effect modification in Caucasians. In terms of baseline values of the main outcome (level of albumin extraction rates), differences between genotypes limit the interpretation of these results. Looking at the results of the Asian study [21], DD carriers did not benefit from ACE inhibitor therapy while DI carriers showed moderate and II carriers showed large treatment responses. Arguably, these conflicting results for treatment success of ACE inhibitors between the genetic subgroups in Asians and Caucasians might be attributed to a different genotype-phenotype relationship. In Asians, the prevalence of the D allele frequency ranges from 27 to 40 percent, whereas in Caucasians, it ranges from 50 to 63 percent. Additional ethnic factors might also affect these genotype-phenotype relationships. For example, the level of circulating ACE is 60% higher in Caucasian DD carriers than in II carriers, whereas for Asians, there are no differences [8,38]

From the patient and clinician's perspective, it is still too early to draw solid conclusions about the optimal treatment among different genotypes. We can, however, speculate that an effect modification exists and that pharmacogenetic testing of the I/D polymorphism might provide additional information about the adequate treatment for these patients. From the public health perspective, it remains unclear whether pharmacogenetic testing would be justifiable in clinical practice. Before investing additional resources to reevaluate our preliminary observations in a primary study of high methodological quality, it might be informative to assess whether screening patients for the I/D polymorphism would have potential economic value. One recent economic analysis showed, for example, that screening men for the I/D polymorphism before starting lipid-lowering therapy with statins would result in considerable cost savings [39]. Thus, estimations of the potential cost-effectiveness of this pharmacogenetic test might be worth considering before starting ACE inhibitor treatment.

## Conclusion

We conclude that evidence is still scarce as there are few pharmacogenetic studies of high methodological quality with comprehensive reporting of their results. Nevertheless we did note a trend towards better response to ACE inhibitors in Caucasian DD carriers compared to II carriers. Future efforts should focus on conducting high-quality pharmacogenetic studies, and reporting of genetic data should be improved.

## Competing interests

The author(s) declare that they have no competing interests.

## Authors' contributions

All authors approved the manuscript.

## Funding disclosure

The study was supported by unrestricted educational grants from AstraZeneca, Switzerland, and from Pfizer, Switzerland. Dr Bachmann's work (grants no. 3233B0-103182 and 3200B0-103183) was supported by the Swiss National Science Foundation. 
